# Hygiene programming during outbreaks: a qualitative case study of the humanitarian response during the Ebola outbreak in Liberia

**DOI:** 10.1186/s12889-020-8240-9

**Published:** 2020-01-31

**Authors:** Alexandra Czerniewska, Sian White

**Affiliations:** Department of Disease Control, Faculty of Infectious and Tropical Diseases, London School of Hygiene and Tropical Medicine, London, UK

**Keywords:** Ebola, Liberia, Hygiene, Behaviour change, Programme design, Theory, Outbreak

## Abstract

**Background:**

Hygiene promotion is a cornerstone of humanitarian response during infectious disease outbreaks. Despite this, we know little about how humanitarian organisations design, deliver or monitor hygiene programmes, or about what works to change hygiene behaviours in outbreak settings. This study describes humanitarian perspectives on changing behaviours in crises, through a case study of hygiene promotion during the 2014–2016 Liberian Ebola outbreak. Our aim was to aid better understanding of decision making in high-stress situations where there is little precedent or evidence, and to prompt reflection within the sector around how to improve and support this.

**Methods:**

We conducted in-depth, semi-structured interviews with fourteen purposively-sampled individuals (key informants) from international organisations involved in hygiene behaviour change during the outbreak. Through thematic analysis we identified the decisions that were made and processes that were followed to design, deliver and monitor interventions. We compared our findings with theory-driven processes used to design behaviour change interventions in non-outbreak situations.

**Results:**

Humanitarians predominantly focussed on providing hygiene products (e.g. buckets, soap, gloves) and delivering messages through posters, radio and community meetings. They faced challenges in defining which hygiene behaviours to promote. Assessments focused on understanding infrastructural needs, but omitted systematic assessments of hygiene behaviours or their determinants. Humanitarians assumed that fear and disease awareness would be the most powerful motivators for behaviour change. They thought that behaviour change techniques used in non-emergency settings were too ‘experimental’, and were beyond the skillset of most humanitarian actors. Monitoring focussed on inputs and outputs rather than behavioural impact.

**Conclusions:**

The experiences of humanitarians allowed us to identify areas that could be strengthened when designing hygiene programmes in future outbreaks. Specifically, we identified a need for rapid research methods to explore behavioural determinants; increased skills training for frontline staff, and increased operational research to explore behaviour change strategies that are suited to outbreak situations.

## Background

In recent decades, a number of factors have led to a rise in the rate of emerging infections with the potential to cause internationally significant epidemics [1–3]. The outbreak of Ebola Virus Disease (the Ebola outbreak) in West Africa between 2014 and 2016 is perhaps the most pertinent recent example of this. Hygiene promotion is a cornerstone of humanitarian response during outbreaks. However, at the start of the Ebola outbreak in 2014, there was limited evidence about what types of hygiene promotion activities were effective in outbreaks generally [4, 5], and almost no evidence about the effectiveness of hygiene promotion in Ebola outbreaks. Despite a lack of evidence and guidance, humanitarians were under pressure to act. This dilemma is not new - the challenges of making critical programmatic decisions with imperfect information has been documented as an area of concern in many humanitarian responses [6–8]. Our aim in this study was to aid better understanding of, and ultimately better support for, decision making in high-stress situations where there is little precedent or evidence.

### Ebola virus disease (EVD) in Liberia

Ebola virus enters the body through orifices, mucous membrane or breaks in the skin. The greatest risk is from direct contact with bodily fluids of a symptomatic or deceased patient, namely blood, vomit, excreta, sweat, saliva, semen, or breast milk. Direct contact with an infected person’s skin is believed to be lower risk, and transmission by contact with the virus on environmental surfaces is rare [9, 10]. The virus is not transmitted through food or water, and is not airborne [10].

**Fig. 1:**
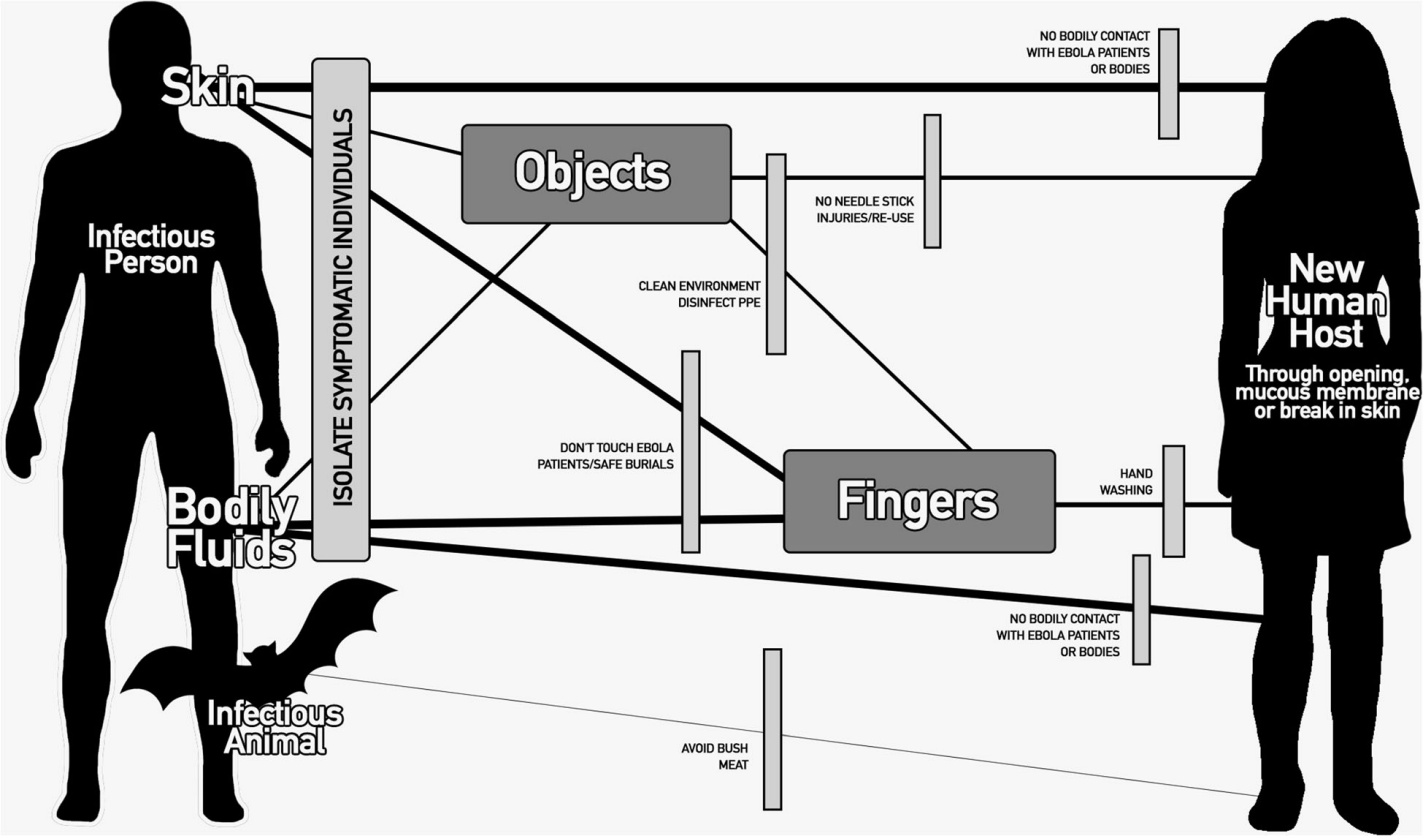
Ebola Virus Disease transmission diagram (author’s diagram, images free to use under creative commons license - CC0 1.0)

The 2014 Ebola outbreak was classified as a ‘Public Health Emergency of International Concern’ because of its high caseload, mortality rates of nearly 40%, and geographical spread (World Health Organisation, 2016b). Previous Ebola outbreaks (in West and Central Africa) have occurred in rural populations and declined relatively quickly. By contrast, in September 2014, the number of new Ebola cases in Liberia was doubling every 10–15 days [11]. With no cure or vaccine yet available, preventing transmission was crucial. The altered ‘F Diagram’ in Fig. 1 depicts the known viral transmission routes from an infected (symptomatic) person/animal to a new (in this case, human) host, based on limited evidence from previous outbreaks with comparable strains [10, 12], laboratory and non-human testing, and biologically plausible evidence from testing on similar viruses [13]. Thicker arrows indicate greater likelihood of transmission through this route. The vertical boxes -show the ‘barriers’, or interventions that can be put in place to stop or minimise transmission. Handwashing with soap is normally considered the main ‘hygiene barrier’ for infection control, but in the context of Ebola, hygiene interventions can be much more wide-reaching, including avoidance of skin-to skin contact with the sick; ‘safe’ burials, cleaning surfaces that could be contaminated by infected human fluids (at home or in health facilities), and properly cooking bush meat.

Before the Ebola outbreak nearly a quarter of Liberia’s population did not have access to water sources that were safe from contamination, half practised open defecation (rising to 68% in rural areas), and only 1% of the total population had access to a handwashing facility at home with soap and water [14].

### Changing hygiene behaviours

Behaviour change is necessary for most public health interventions. Until quite recently the status quo in public health interventions has been to educate populations about the health risks and benefits of different behaviours. A growing number of studies now suggest that there is little association between knowledge of health benefits and actual performance of a behaviour [15–19], especially when compared to other factors that determine behaviour [20, 21].

A contrasting approach to behaviour change programming is to create an intervention that targets key determinants to shift behaviour. This is done after thoroughly assessing all of the factors that positively or negatively influence behaviours in a particular context (hereafter we call these behavioural determinants). Instead of improving knowledge, this approach might highlight the need to change social norms, appeal to specific personal motivations, or introduce behavioural cues into the environment. Michie et al. [22] identify 93 distinct techniques like these, which can be used to cause a change in behaviour. Interventions designed in this way are often called ‘theory-driven’ since they draw upon a theoretical understanding of how behaviour is determined and changed. They are usually accompanied by a testable hypothesis of the precise mechanisms by which behaviour is expected to change (a theory of change). Interventions developed through theory-driven approaches have been able to demonstrate substantial shifts in observed hygiene behaviour in non-outbreak settings [23–27], but we know little about whether theory-driven approaches are feasible or effective in emergency settings, or how similar or different the determinants of behaviour change are. For example, communicating health risks during infectious disease outbreaks may be more effective than in non-outbreak settings [28, 29], but may only have short term effects [30]. Using motives like disgust, shame or nurture (the desire to do what is best for your child) might be just as relevant in emergencies, but there may also be additional ethical considerations of using such motivators with vulnerable communities [31, 32].

Frameworks and processes exist to help programme designers to select the most appropriate behaviour change approaches for each problem and context. Within the water, sanitation and hygiene (WASH) sector the following frameworks provide both definitions of determinants and a process for programme design: RANAS (Risks, Attitudes, Norms, Abilities, and Self-regulation) [33]; Designing for Behaviour Change [34], and Behaviour Centred Design [35]. There is increasing interest in designing theory-driven and evidence-based interventions to improve hygiene promotion in major disease outbreaks or other emergency settings [30, 32], but so far there has been little attempt to translate existing approaches. The exception is the RANAS questionnaire, which has been used to identify behavioural determinants (retrospectively) during the 2010 Haitian earthquake, in drought-affected Ethiopia, and with the aim of preventing an Ebola outbreak in Guinea-Bissau [36–38].

Although the terminology and methods vary, the process for designing interventions is relatively similar across these frameworks. Each proposes a five-stage, theory-driven programme design process. The first stage typically involves programme designers gathering existing knowledge about the target behaviours, audience, and context (in this paper we call this stage ‘assessment’). Deeper insights are then gathered by carrying out formative research to assess the behavioural determinants for that context (here called ‘understanding behaviour’). The third stage involves iteratively creating and pre-testing the intervention package (here called ‘programme design’). ‘programme delivery’ and ‘evaluation’ are stages four and five. Utilisation of an intervention design process, like this, is becoming increasingly commonplace in the development sector.

### Aims and objectives for the current study

Our study aimed to understand decision making in high-stress situations where there is little precedent or evidence, and ultimately prompt reflection within the sector around how to improve and support this. Our study objective was to describe humanitarian perspectives on changing behaviours in crises, through a case study of hygiene promotion during the Liberian Ebola outbreak. This study does not attempt to answer the question of what worked or did not work to change hygiene behaviours, but we do report participants’ perceptions of successes, constraints, and the feasibility of alternative approaches.

## Methods

### Sample selection

We conducted in-depth, semi-structured, key informant interviews during July and August 2016. Participants were individuals from international organisations that implemented, funded or were otherwise involved in interventions to change hygiene behaviours in communities or in healthcare settings during the Ebola outbreak in Liberia (2014–16). We included bilateral donors, implementing partners and technical experts.

Participants were purposively sampled. We contacted all organisations from an initial list of 22 organisations identified through a review of minutes of the WASH Cluster and Case Management committee meetings of the National Ebola Taskforce in Liberia (organisations working in Liberia typically attended these meetings). Interviews were requested by email, with three unanswered emails considered as a refusal. Participants were asked for further recommendations of organisations and individuals to approach. Eligible participants had worked on the Ebola response for all or part of the time between March 2014 and the outbreak’s declared end in June 2016 [39]. Eligible organisations had to be involved in designing or delivering hygiene interventions in programmes in the community or in routine health facilities.

### Data collection

We prepared a list of interview questions to guide our interviews, which could be adapted flexibly during each interview (see Additional file 1). We asked participants to describe the interventions they were involved with, and how decisions were made during the design, delivery, and monitoring of interventions, i.e. across the five stages of the theory-driven programme design process described above. We also asked participants about their perceived program constraints and successes. All interviews were conducted by the lead researcher (AC) in English, recorded and transcribed.

### Data analysis

We analysed data concurrently with collection, using a thematic, six-stage approach suggested by Braun and Clarke [40]. Concurrent analysis of the transcripts helped us to identify when we had reached a point of saturation. We coded transcripts inductively using NVivo 11 software, and themes were grouped under headings based on the five stages of theory-driven programme design process. In the discussion, we reflect on the differences between the approach described to us, and approaches taken in non-emergency settings, and make recommendations about how we can more effectively design, deliver and monitor hygiene programmes during outbreaks and crises, when compromises have to be made.

### Ethics

Participants were provided with an information sheet explaining the purpose of the study, the voluntary nature of participation, and our commitment to preserving confidentiality and anonymity of individuals and organisations in reporting. All participants were adults who gave written consent after an opportunity to ask any questions they had. Ethical approval was obtained from the London School of Hygiene and Tropical Medicine (Ref. 11,349) and the National Review Ethics Board of Liberia (Ref. NREB-015.06).

## Results

**Fig. 2:**
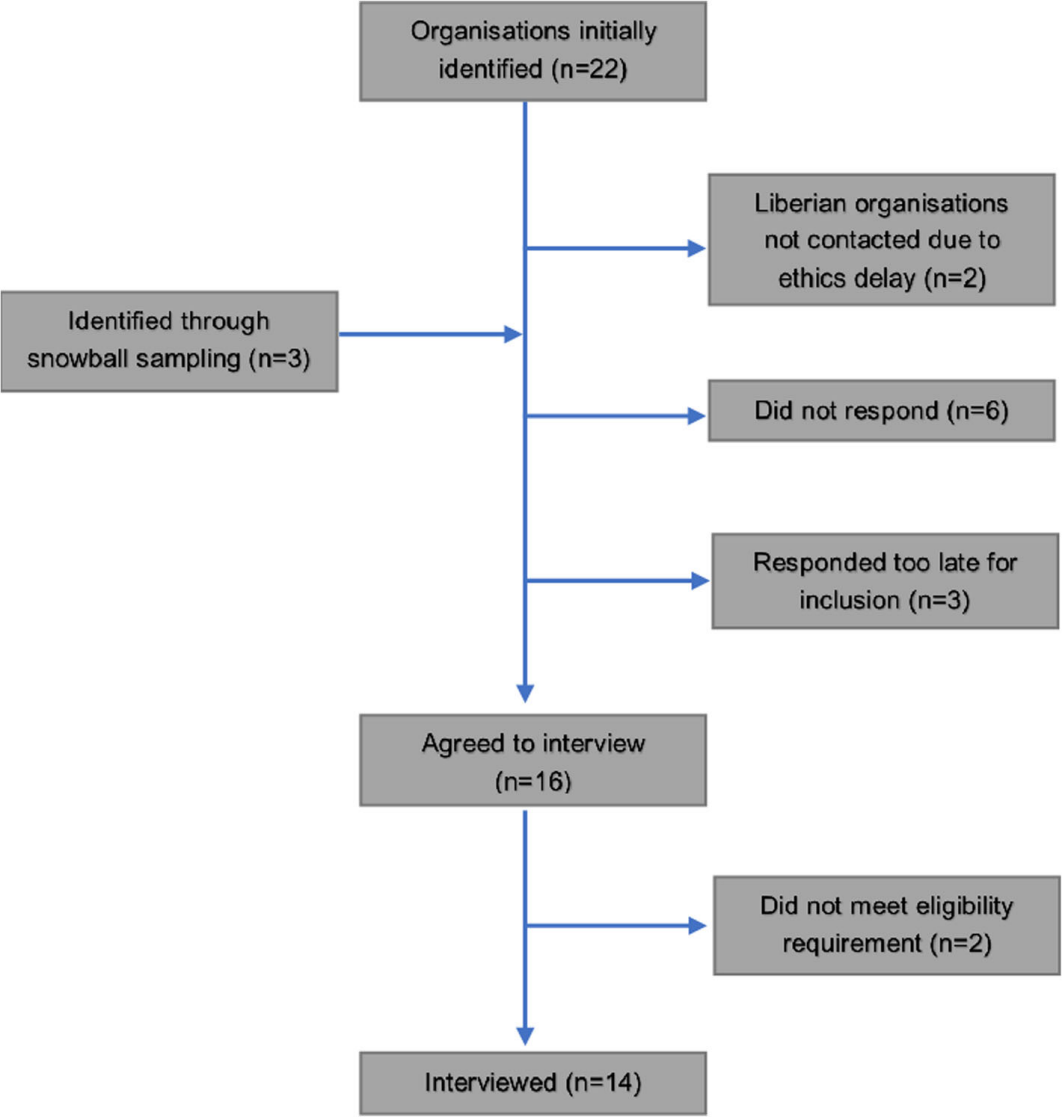
Description of sampling and participation rates

We conducted in-depth interviews with 14 individuals from 12 international organisations. Nine were from international non-Governmental organisations (iNGOs) three from organisations in the United Nations (UN) system and two from bilateral donor agencies. Three organisations agreed to interviews but responded too late for inclusion and six organisations did not respond (Fig. 2). These organisations were not further pursued because the authors felt that a degree of saturation had been reached through the initial 14 interviews.

All participants were at a senior level (Programme Manager or higher) and had between six and 24 months’ experience working on the Ebola response in Liberia. Seven participants described themselves as Water Hygiene and Sanitation (WASH) or Infection Prevention and Control (IPC) specialists, one as a communications specialist, and six had oversight over several different areas. Twelve interviews were conducted by telephone or Skype, one face-to-face and one participant requested to respond by email. On average, interviews lasted around 1 h (range 45–90 min). Four participants focused on behaviour change within routine health facilities (named H1 - H4) and ten in community settings (C1- C10). All but one participant stated changing hygiene behaviours as a key organisational priority.

Handwashing was the most commonly mentioned hygiene behaviour, but emic definitions of ‘hygiene interventions’ were much wider-ranging than we had anticipated. Participants often described behaviours such as reducing general physical contact (e.g. not shaking hands); keeping a distance from sick or deceased individuals (and their bodily fluids), and ensuring burials were conducted by trained burial teams. Wider aspects of infection control, water provision and sanitation were also described as being part of hygiene programming. We therefore include these elements in our analysis. Our results are structured according to the five stage, theory-driven programme design process employed in non-emergency settings.

### Stage one and two: assessment and understanding behaviour

The main focus of early assessments carried out in communities or health facilities was to identify gaps in infrastructure (e.g. wells, or road access) and to make contact with local leaders who could facilitate access to communities. None of the participants reported systematically collecting information about current hygiene behaviours. One participant reported that emergency responders did not attempt to learn from existing hygiene promotion interventions in Liberia.

*A whole raft of new emergency staff entered Liberia and tried to work in an emergency vacuum. They assumed there was no existing data and did little to try and understand the previous situation […] it could have been very useful to look at existing research and materials that had been developed. No emergency staff from INGO emergency teams or government staff showed any interest in that. [C7].*

Several participants explained that it was seen as a low priority to understand psychological or social determinants of hygiene behaviour determinants, because it was decided early on that hardware and infrastructure would take precedence over behaviour change ‘software’ (e.g. training or messaging):

*It’s all very well going in and doing [hygiene] training but if they haven’t got pit latrines and they haven’t got a clean water source it’s a bit like Marie Antoinette and ‘let them eat cake.’ [C2].*

*I always get frustrated with social and behavioural sciences […] the idea that people aren’t doing things because they are not educated or they lack information […] behaviour change sinks in when the materials are actually there. [C3].*

Many of the hygiene behaviours recommended were not precisely defined, i.e. in terms of who should perform the behaviour, when it should be performed, or how. Some participants felt that this resulted in weak messages:

*The handwashing messages were terrible. ‘Wash your hands all the time’ seemed to be the primary message. There was no information about when, or how to wash hands or who should be doing it. [C7].*

The lack of evidence and precedent in the early stage of the outbreak made it particularly difficult for responders to be more precise about the behaviours required to reduce disease transmission. For example, one major point of contention early on was whether to advise handwashing with soap or handwashing with a chlorine solution:

*We were so worried that we didn’t have any evidence; we didn’t know whether hand washing with soap or chlorine was better...Technical experts were going in circles because they didn’t have [academic] papers to look at, and the ones that were there were fuzzy. [C4].*

### Stage three: Programme design

#### The intervention components

Providing water, buckets with taps, soap/chlorine, gloves and other protective clothing in communities and health facilities was reported as the biggest component of the hygiene interventions. Hygiene messages were delivered alongside this on posters, billboards, on radio programmes, and face-to-face, by local Community Health Workers (CHWs) and local volunteers across the country throughout the outbreak. The nature, intensity and coverage of this community engagement varied across the country. This depended on the effectiveness of existing community health systems and county-level emergency operations centre, as well as the strategy of the different international organisations supporting the government in that area.

**Fig. 3:**
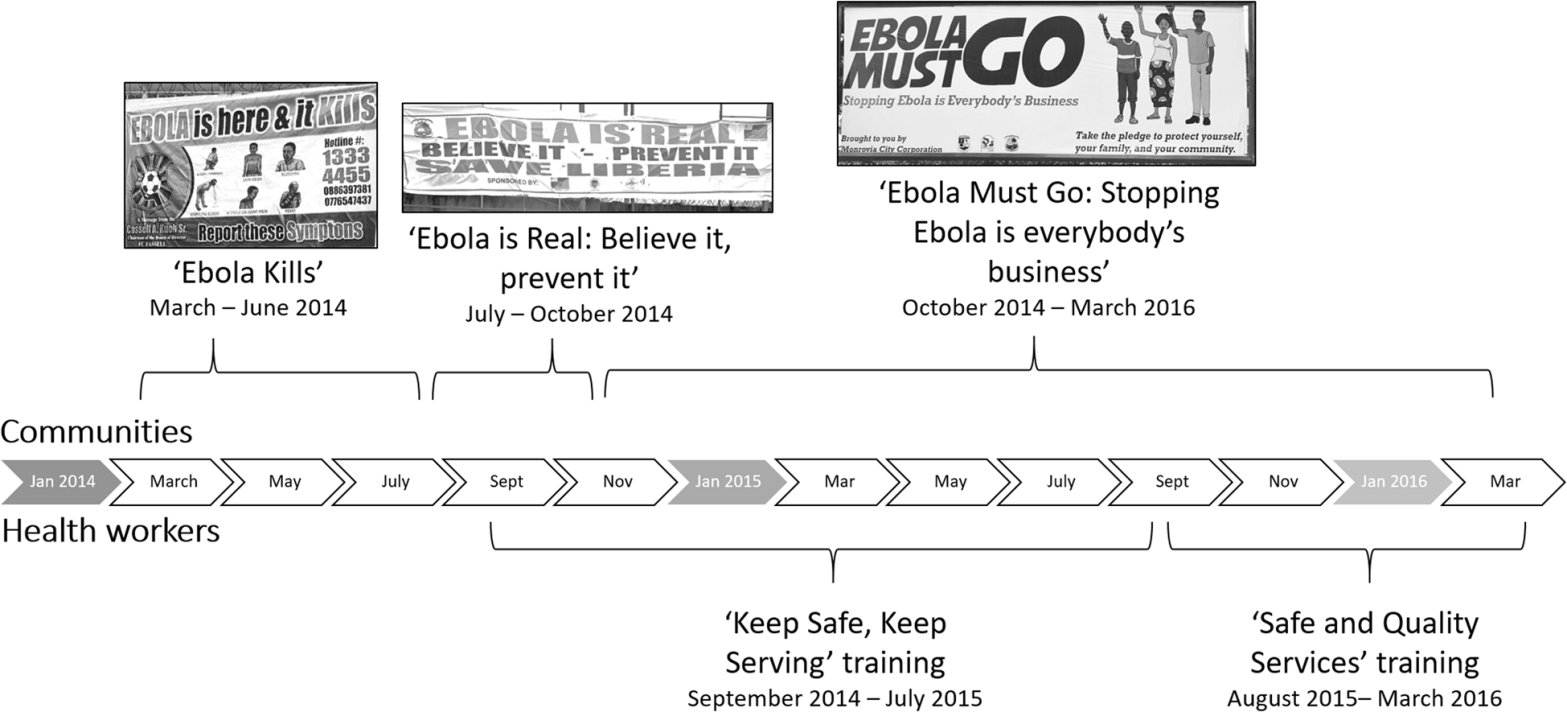
Timeline of national community campaigns and health worker training packages (author’s diagram, the images are photographs of billboards developed by the Liberian Ministry of Health and other Non-Government actors)

The messages evolved over the course of the outbreak, and broadly aligned with the headline messages chosen by the government and key media partners. The first headline message - *‘Ebola Kills’* – ran from March 2014 until around June. Hygiene messages in this phase aimed to amplify a feeling of fear and risk of death if you did not perform the recommended hygiene behaviours. This was replaced by *‘Ebola is Real’* from approximately July to October 2014, where hygiene messages still focussed on amplifying perceived risks of transmission but with more of a focus on understanding the disease without evoking fear. Finally, the *‘Ebola must Go’* message encouraged the maintenance of behaviours to protect family and country. Figure 3 describes the phasing of these messages throughout the outbreak. The creative process by which the slogans, images and hygiene messages were chosen and produced was reportedly driven by the Government of Liberia with support from a small number of other organisations. Most participants were positive about the campaigns created and the delivery channels used to disseminate them.

Two training packages were targeted at health workers in routine health facilities. The *‘Keep Safe, Keep Serving*’ training package was developed in early September 2014 to address issues related to triage and isolation, cleaning and waste management, protective clothing, and handwashing. From August 2015, the ‘*Safe and Quality Services’* training package replaced this – emphasising that health workers should understand general infection control and hygiene principles and be able to apply them to protect themselves and patients against many diseases. This package also introduced psychosocial training modules. Both were developed by small working groups of partners.

### Decisions and processes affecting how interventions were designed

Participants described the compromises that were made to a typical intervention design process due to the nature of the emergency context:

*The typical approach is you scope it, you have your concept, your expert panel groups, you create it, you pilot it, and then disseminate it. 5 stage approach. When you think about the Ebola context, the [health worker training package] was literally made in a couple of days by three people locked in a room [...] The biggest difference [in a non-emergency setting] is the time they have to create this and the loops and hoops they jump through to make sure it is relevant, acceptable, and endorsed. With the epidemic stuff it was very much ‘who cares? Roll it out, looks good on paper, go.’ [H1].*

*It was a true time of crisis and it got out of control before they knew it and there was not a lot of time to think about the approach to how to get people to do the right behaviour. It became ‘this is what you have to do’; it was not ‘how do we do this together to get the right outcome’. […] Right or wrong, that’s just what happened. [H3].*

These compromises reportedly led to some issues with implementation. For example, the messages delivered on posters and by health workers aimed to improve biomedical knowledge and understanding of transmission pathways, based on an assumption that people would modify their behaviour once they knew what was ‘best’ for them.

*In an emergency, information is probably the most important thing you can give people because then you can make a well-informed decision […] people will generally act in their own best interest. [C9].*

However, some participants reported that this medicalised view of disease was at odds with the way many communities conceptualised health and the body:

*In some of the extremely remote settings, those messages of ‘Ebola is Real’ might not have even been internalised and understood because of really strong traditional beliefs in other spiritual things. [C3].*

*[Liberian people] have a huge element of understanding a disease to be spiritual. You have to come in with some kind of understanding. [...] You can’t go in with a biological teaching because they have no basic concept of biology. [C2].*

All emergency responders were expected to use the same, approved hygiene messages in their promotional materials. These were decided by the Government of Liberia with selected international partners who would instruct other organisations at a weekly ‘messaging and media’ working group. Most participants agreed it was important that key national messages changed simultaneously across the country.

*We don’t want everybody doing something different because that will just breed confusion. [C4].*

*We had to change at the same time […] The old messages and jingles had to be removed so we are all on the same planet, same page. [C8].*

Others reflected that the decision to take a standardised approach had hampered the effectiveness of the messaging in some areas where it was not reflective of the local situation and needs:

*I don’t think the messages, as they were, were really that effective. Different pockets of the population had different exposure to Ebola. In Lofa [county] you could say ‘Ebola is real, don’t touch dead bodies’, right, yeah. I’ve seen that happen. But there are villages down in Rivercess [county] that have never had any cases. [C9].*

When it came to deciding on emotional or psychological elements of hygiene messaging, the fear of contracting Ebola was assumed to be the most powerful motivator of behaviour change:

*Fear played a very big role. Everyone was afraid; if I don’t do it [handwashing] I’m going to die. [C1].*

*As people and communities came round to the understanding that Ebola was real … and they saw people getting sick then they did start to change their practices. So I think there was a level of fear that drove that. [C2].*

However, participants noted that motivation via fear sometimes produced behaviour change that was unexpected, unproductive or even dangerous:

*At first the message was ‘Ebola Kills’ so everybody was … waking up saying ‘oh my god - there is no alternative so why are you telling me even to wash my hands?’ So we had to change after listening to the communities and realising that they had understood the message but the fear feeling was not going to work because it was not supporting the follow up actions. [C8].*

*Because of the fear, people were putting a lot in the water – Dettol, chlorine, soap because they knew this would stop the virus. The outcome was people getting their hands irritated and that was going to cause more problems. [C8].*

Messages rarely went beyond increasing perception of risk and the motivation of fear. In retrospect, some participants reported this as a missed opportunity, suggesting that factors such as peer pressure or shame may have also been important:

*I think there was a huge missed opportunity to use other determinants and messages, considering that we know fear only works when there is a threat of the public health issue. [C7].*

*‘I think the best way to reach [health workers] is by speaking a language they’re going to understand and connecting a message that actually tugs at their heartstrings. Otherwise I don’t see why anyone would be motivated to do anything differently. [H7].*

*I think there’s strong human behaviour of peer pressure and if you see everybody else doing it then you’re going to be like, ok well I should do this too. [C3].*

However, two organisations were reluctant to use negatively framed motivators like shame:

*The public shaming piece is not something we would ever want to condone; however, we have seen that there is a lot of evidence that it is successful in many settings including here in Liberia. We would usually take more of a compassionate approach. [C3].*

When asked about the processes used to design the behaviour change components of hygiene programs, participants answered with less fluency (and more pauses) than in other parts of the interviews. Behaviour change as was generally perceived as a specialised skill:

*I by no means claim to be an expert on any of this. [H3].*

*I’m not a specialist in this area. [C9].*

*I don’t know in terms of individual behaviour change theories. We do have a cadre of workers who are behaviour scientists. […] but I’m just not engaged enough. [H1].*

Employing alternative behaviour change techniques (other than improving knowledge and increasing perception of risk) was considered to be too ‘experimental’ by some respondents:

*It was all about saving lives. There wasn’t much time to do a lot of experiments. [C1].*

### Stage 4: Programme delivery: humanitarian perspectives about what worked and was acceptable

Participants felt strongly that the way interventions were delivered was the key to their success in changing behaviours. For example, there was a belief that hygiene messages would be more effective if the ‘right’ people delivered the messages and interventions. They avoided deploying ‘outsiders’ for this task, who would not have the communities’ trust, and instead used locally known and trusted messengers:

*One thing that was critical was having Liberian healthcare workers talking to Liberian healthcare workers, and they said ‘look, we went through the same thing you’re going through, you almost died, we almost died, if we all just do this together and do what we’re supposed to do, we won’t get Ebola.’ [H4].*

*We had about 450 staff and they were local staff. It was them that did all the training so that it was culturally appropriate, language appropriate and the community was receptive to it. [C7].*

Senior leaders from local and central government, and religious institutions, were also seen as very important agents to create change. Some participants attributed this to the strong culture of ‘top-down’ authority systems in Liberia.

*We worked through community leaders, religious leaders, and traditional leaders because the community tends to listen more to its community leaders. [C6].*

*If the chiefs are saying ‘you need to do this’ it’s more likely that people will do it. If the chiefs and MOH were saying the same thing that was the double whammy. [C9].*

Several participants said that an approach of engaging audiences in a ‘dialogue’, or two-way conversation, about hygiene behaviours was important for creating behaviour change because this increased acceptability and helped humanitarian actors to adjust messaging to local circumstances:

*The whole idea was not to go in with a pre-established mechanism or approach. It was more to listen to the community, try to pick out the things that they’re most concerned with … this is probably the best way to do it. [C4].*

*Lots of alterations were made along the way as the [NGO] staff interacted in the communities, with reactions to the messages. Adjustments were made in the language, adjustments were made in the approach and so on. [C10].*

### Stage 5: evaluation

Monitoring and evaluation were primarily designed to identify regions of particular concern – for example communities where there was resistance and denial, or health facilities with very poor capacity for infection control.

Organisations monitored inputs and activities rather than outcomes or impact. In communities and health facilities indicators were collected on provision (e.g. numbers of buckets distributed) and reach (e.g. estimations of those attending awareness events). Other data was anecdotal, e.g. through observing training sessions or collecting ad-hoc feedback from communities. None of the organisations interviewed systematically monitored outcomes related to hygiene knowledge, awareness, or behaviour.

*We generally have some basic M&E from the beginning in terms of how much we have distributed because that’s easy to record, number of buckets. We were able to estimate the number of people we reached with Ebola messaging, how well that worked I have no idea. [C2].*

*[We were] not watching healthcare workers for X number of hours interact with people […] I think that’s a big weakness, it turns into an anecdotal situation, which is very common in the response throughout. [H1].*

In Table 1 we summarise our key findings to the theory-based model of hygiene programme design.

**Table 1:** Description of the 5 theory-based steps used in standard programme design compared to our findings of what was done during the Liberian Ebola outbreak

Theory-based steps for designing a hygiene behaviour change programme	Process of designing hygiene behaviour change programmes in Liberia during the Ebola outbreak.
Assessment - programme designers gathering existing knowledge about the target behaviours, audience, and context	Existing research and resources on hygiene behaviour were not utilised.
	In the absence of clear evidence humanitarians struggled to define key hygiene behaviours.
Understanding behaviour – formative research is undertaken to develop a deeper understanding of behaviour at the current time	Assessments focused on the availability of infrastructure rather than behavioural barriers or determinants.
Programme design - iteratively creating and pre-testing the intervention package with your target audience.	The design process was rushed with actors feeling the need to act.
	Programmes primarily focused on providing hygiene products and teaching people about Ebola transmission and preventative behaviours.
	Hygiene messages were standardised across the country. This was viewed as minimising risk and confusion but it also meant that messaging was often not contextualised to different experiences within the country.
	Opportunities may have been missed to utilise alternative behaviour change techniques, particularly emotional or psychological determinants of behaviour.
Programme delivery – training and supporting staff as they delivering activities as intended.	Programmes were seen as more successful when they used ‘trusted messengers’ and created a dialogue with community members.
Evaluation – process and impact evaluation of the project.	Monitoring primarily focused on identifying regions where there was community denial or health facilities infection control.
	Organisations monitored inputs and activities rather than outcomes or impact.
	There was no systematic monitoring of hygiene knowledge, awareness, or behaviour

## Discussion

The Ebola outbreak of 2014–16 was unique in its scale and complexity. There was no ‘roadmap’ for the design of hygiene behaviour change interventions in such a context. However, the decisions made during this outbreak have informed the humanitarian response to the subsequent 2018/19 Ebola outbreak in the Democratic Republic of the Congo and have had wide-reaching impacts on global public health landscape [41]. As with any emergency of this significance there has also been substantial criticism of decision making during the outbreak and identification of areas where learning could have been enhanced [42, 43].

We did not set out to criticise the interventions created, but to understand how hygiene promotion interventions were designed, delivered and monitored by humanitarian response organisations in a difficult situation with limited evidence or precedent. This was done with the aim of prompting greater reflection within the emergency response sector about how hygiene programmes can be designed and delivered more effectively, and meet the challenges of future disease outbreaks with improved tools and approaches.

By coding the results under the five stages of ‘theory-driven’ programme design, we identified differences between the processes used during this Ebola outbreak and the methods developed for designing, implementing and evaluating behaviour change interventions in non-outbreak settings. Vujcic et.al [44]. documented humanitarian perspectives on the design of hygiene programs for displaced and refugee populations. Among other things they identified challenges in the contextualisation of programming, limited local capacity on behaviour change and poor monitoring of hygiene behaviour change programs. We also identified significant barriers to implementing theory-driven behaviour change interventions in outbreaks. Three barriers in particular are discussed here, each highlighting the need for sector wide reflection and discussion:

### Limited attention to behavioural determinants

Criticism of the international response to the Ebola outbreak frequently focused on the failure to engage communities early on, and poor alignment to local context and culture [45–47]. We found that participants had given this a lot of thought in the delivery phase, but not in the design phase. There was no systematic attempt to collate existing knowledge on behavioural determinants or build on this knowledge through further research. Consequently, the interventions that emerged focused relatively narrowly on the provision of ‘hardware’, improving knowledge, and increasing fear. These interventions might have been effective at reducing transmission [48]. Evidence from stable settings certainly supports the initial choice among many humanitarian organisations to prioritise infrastructure [49–52]. Provision of handwashing infrastructure has also proved effective in protracted crises and cholera outbreaks, but in these contexts was strengthened by a ‘soft’ behaviour change-focused intervention [53, 54].

Interventions emphasising the biomedical risk and amplifying fear appeared to be poorly accepted by some communities - in certain cases leading to harm e.g. skin irritation caused by excessive use of chlorine. The authors of the RANAS theory of behaviour argue that it is common for health promotion messages in emergency settings to focus on increasing the ‘*perceived susceptibility and perceived severity of contracting a disease, and factual knowledge about the possibility of being affected’* [33] p.561, forgetting other behavioural factors that are also important predictors of behaviour. Other studies have even shown that behaviour can change during disease outbreaks without people feeling any increased sense of infection risk [55].

Some participants felt there had been a missed opportunity to explore a wider range of social determinants of behaviour, particularly social pressure and social norms. Reviewing posters and radio messages from the outbreak suggests that these determinants were already being targeted to some extent (for example*: ‘Ebola must Go. Stopping the spread of Ebola is everybody’s business’*) [56], but that they could have been utilised more effectively.

Formative research tools to help responders to identify a wider range of behavioural determinants could be developed for emergency settings. Participants in this study felt that behaviour change was beyond their remit and skills, suggesting that new tools should be accessible to non-specialised practitioners. Vujcic, Ram [32] identified a similar knowledge gap in humanitarian emergencies and recommended training in behaviour change theory for staff at every level.

There was huge international pressure to respond rapidly at the start of the Ebola outbreak, which prevented the often lengthy process of collecting and analysing formative research data. New formative research tools must also be able to generate results quickly, and must be useable at any stage of an outbreak, for example to allow emergency responders to adjust their programmes based on the findings once the initial emergency infrastructure is in place in the acute phase of an outbreak.

Participants expressed wariness to ‘experiment’ with new types of interventions, or use motivators like shame or disgust to encourage behaviour change, both of which have proven useful for behaviour change in non-emergency contexts [57–60]. DuBois et.al [45]. argue that responders to the Ebola outbreak were disincentivised to test novel behaviour change interventions because ‘rational’ education and information campaigns are easier for donors and the international press to understand [45]. A possible way to incentivise or create demand for new interventions is to change perceptions around ‘experimenting’ e.g. using methods that have already gained traction such as ‘social learning’ methods [61], or approaches that involve working more closely with communities [62–64]. Small scale pilots of different approaches are needed to generate more evidence of what works in emergencies and outbreaks. Such initiatives should involve donors and practitioners who can translate experiments into scalable interventions if successful.

### Limited questioning of assumptions

We identified assumptions about the design and delivery of behaviour change interventions in this emergency setting, which are not based on evidence and could benefit from further research. For example, most participants believed firmly that nationwide uniformity of key messages (on posters and radio) was important to avoid confusion and encourage behaviour change. Similar findings have been reported in other emergency settings [44]. We suggest that the real reasons might be related to a desire to demonstrate strong leadership in a time of crises, and that more research is needed into uniform versus tailored messaging before this becomes accepted as standard practice in future outbreak emergencies.

A second example is the assumption about local capacity. Participants indicated a preference for messages or other interventions to be delivered by local staff who had a deeper knowledge of the local context. However, being local does not necessarily mean you have the natural ability to promote behaviour change effectively, or to make the best decisions about how to tailor interventions on the ground. We suggest that interventions that rely on interpersonal communication need to build in measures to train staff at all levels, and to monitor, document and share field-based changes to improve practice.

### Difficulty of monitoring and evaluation

Participants described the monitoring of inputs (e.g. numbers of buckets purchased) or outputs (e.g. meeting attendance or communities visited). However, most respondents felt unable to assess the impact of their programme on behaviour. We were unable to identify other literature documenting the impact of Ebola hygiene programming on behavioural outcomes, but there is some evidence from other studies that hygiene promotion efforts were successful in increasing knowledge about Ebola symptoms, transmission and ideal hygiene practice [65–67]. However, we know that knowledge is rarely sufficient to change hygiene behaviour [57, 68, 69], and these studies document that hygiene programs were less effective in changing deep seated beliefs or sustaining change over the full course of the outbreak [70].

It is difficult to get valid and reliable health or behaviour change outcomes in emergency settings [5]. However, a first step would be to align emergency response work with standardised indicators used in the development sector e.g. the Joint Monitoring Programmes’ handwashing proxy indicator [71]. Programs could also include other impact measures, such as behavioural observations, at a small scale. This would help build a stronger picture of intervention effectiveness and the mechanisms of change.

### Limitations and reflexivity

Interviews were conducted with a small number of mid- and senior-level staff from international organisations. Results therefore omit important local voices from government, or staff more heavily involved in day-to-day engagement with communities. Approval to interview representatives of the Liberian government or local NGOs was granted too late, and therefore these important voices were omitted from this research. The opinions of Liberians in relation to both the outbreak and the mode of international response have been reported elsewhere [61].

Most participants were willing to discuss their approaches to behaviour change openly during the interview, but some seemed less comfortable to express personal views that deviated from the ‘official’ position of their organisation. Most interviews were conducted by Skype call, but the one participant who answered via email will have had more time to reflect on the questions. Responses may be biased due to recall, or shaped by the large amount of analysis, commentary and criticism the subject had already received.

The lead researcher had existing professional relationships with 10 of the 14 participants, and this ‘insider’ status will have affected the collection, analysis and interpretation of the data.

## Conclusions

This study describes humanitarian perspectives on changing behaviours in crises, through a case study of hygiene promotion during the Liberian Ebola outbreak. We documented how hygiene promotion interventions were designed, delivered and monitored by humanitarian response organisations during the Ebola outbreak in Liberia. We aimed to prompt greater reflection within the emergency response sector about how hygiene programs can be designed and delivered more effectively during major outbreaks. We identified several important practice and knowledge gaps, including the need for rapid research methods to explore behavioural determinants, and increased skills training for frontline staff. We highlighted a number of unchallenged assumptions which act as barriers to exploring alternative behaviour change strategies. Sector-wide reflection and discussion is needed to examine the assumptions and choices that are made by emergency responders, along with increased operational research to ensure we have the right tools and approaches to meet the growing challenges of emerging disease outbreaks.

## Supplementary information

**Additional file 1.** The interview guide used for the semi-structured interviews conducted.

## Data Availability

The datasets generated and analysed during the current study are not publicly available because this could result in identification of participants who gave interviews on the condition of anonymity. Data are available from the corresponding author on reasonable request for researchers who meet the criteria for access to confidential data.

## Abbreviations

(i)NGO: (International) non-Governmental organisation
CHW: Community Health Worker
EVD: Ebola Virus Disease
IPC: Infection Prevention and Control
WASH: Water Hygiene and Sanitation
