# Supplementary material for: Hygiene programming during outbreaks: a qualitative case study of the humanitarian response during the Ebola outbreak in Liberia
Source: BMC Public Health. 2020 Jan 31;20:154. doi: 10.1186/s12889-020-8240-9 (PMC6995147; doi:10.1186/s12889-020-8240-9)
Supplement: Supplementary file 1 — Additional file 1. The interview guide used for the semi-structured interviews conducted. [file 12889_2020_8240_MOESM1_ESM.docx]

**Semi Structured Interview guide**

**This guide is intended to be used flexibly and adaptively depending on the interviewee.*

| **Background details (collected by email or during the interview)** | - Interviewee details [role, organisation] - What hygiene behavior change activities was [organization] involved in commissioning/implementing during the Ebola outbreak in Liberia? [Please list all activities taking place outside of designated Ebola Treatment Centers. E.g. in communities, regular health facilities]. - For each intervention/activity:   - What behavior did the intervention aim to change?   - Description of hygiene intervention   - Target regions/populations   - Role – implementer/funder   - Who designed the intervention?   - Dates of hygiene activities   - Estimated scale (££ and people) - Was the focus of hygiene behaviour change intervention to stop Ebola transmission, or broader health outcomes? - Was the intention for the behavior change be sustained beyond the Ebola period? - What other work was your organization involved with during the outbreak? How much of a priority was this hygiene behavior change work in relation to these other areas of work? |
| --- | --- |
| **Stage 1: Assessment an understanding behaviour** | - What information did [organisation] have on hygiene behaviours in the target areas before Ebola (e.g. baseline data, anthropological records)? - How did you select the behaviours to target? - How was behaviour change expected to occur? - What (if any) is the underlying theory in [organisation] about how behaviour change happens/is driven? - How was this process of assessment different to hygiene programmes delivered by [organisation] in other contexts, if applicable? |
| **Stage 2: Building on knowledge** | - Before designing interventions was any formative research or consultation carried out in the communities/in health facilities by [organisation]? - If so, please describe this formative research   - Type   - Locations   - Target audience   - Duration   - Reports or theories of change produced - If not, was information sought from experts or representatives? - What was the primary purpose of formative research? [e.g. To understand drivers of behavior change, gain acceptance in the community, to consult with local leaders] - If [organisation] delivers hygiene behaviour change programmes in non-Ebola contexts, how did this formative stage differ? |
| **Stages 3 &4: Creating and delivering the interventions** | - What constraints did you have in designing a behaviour change campaign (e.g. cost, time, acceptability etc.)? - How did these constraints effect your decision making? - Describe the interventions designed   - Key messages   - Target audience   - Promotional material (leaflets, videos, theatre?)   - Who delivered the training?   - Were ‘kits’ used? What was in them?   - Was there a field guide? - How did the intervention designed differ from the intervention delivered? - *Hand washing interventions only:*   - What designs were considered and chosen for hand washing stations? (Why were basic buckets with taps often selected?)   - When (i.e. at what key times) were people encouraged to wash hands? - What elements of the intervention were considered most important to achieve effective behavior change at the design stage? - What would you consider most effective now, with hindsight?   - If not mentioned, ask about importance of each of the following:     - Community-led     - Training from external teams     - Ongoing visits or supervision     - Excellent facilitators     - Provision of infrastructure, equipment and supplies     - Information on how Ebola is transmitted     - Triggering of negative emotions like fear     - Other motivations such as nurture, disgust, affiliation?     - Fun or interesting interventions     - Enforcement - If organisation delivers similar programmes in other contexts, what, if anything, was striking about the ways these interventions were (or should have been) created or implemented in the Ebola context in comparison? |
| **Step 5: Monitoring and evaluation** | - Do you think the intervention(s) was effective to achieve behaviour change? - What indicators, if any, were collected and reported on? - Based on evaluation or information from implementers, how did the interventions change over time? - What was different about evaluation during Ebola compared to other contexts? - Do you think the interventions will be effective now Ebola is at a very low level again, or were they purely for the Ebola period? - Given experience in other contexts, was anything surprising about what or was not effective in this setting? |
| **If Ebola returns…** | - If Ebola returns, is hygiene behavior change a key part of the response again? Which behaviors will be the most important to target? - If another Ebola outbreak was to occur what would you do differently in terms of the process you used for designing the behaviour change intervention and its implementation? - At a national level in Liberia, could more have been done to improve hygiene behaviour change? - What was the role of the funder and how could this be improved? |
